# Supplementary material for: Resolving the full spectrum of human genome variation using Linked-Reads
Source: Genome Res. 2019 Apr;29(4):635–45. doi: 10.1101/gr.234443.118 (PMC6442396; doi:10.1101/gr.234443.118)
Supplement: Supplemental Material [file supp_gr.234443.118_Supplemental_longranger-2.2.zip › longranger-2.2.tar.bz2!/lr2.2-public/lib/stan/cmdstan/stan/doc/api/html/likely_8hpp_source.html]

Stan: src/stan/meta/likely.hpp Source File


|  |  |
| --- | --- |
|  | Stan  2.5.0  probability, sampling & optimization |


- Main Page
- Related Pages
- Namespaces
- Classes
- Files

- File List
- File Members

All Classes Namespaces Files Functions Variables Typedefs Enumerations Enumerator Friends Macros Pages

- src
- stan
- meta

likely.hpp

Go to the documentation of this file.

1 #ifndef STAN\_\_META\_\_LIKELY\_HPP

2 #define STAN\_\_META\_\_LIKELY\_HPP

3

4 #ifdef \_\_GNUC\_\_

5 #define likely(x) \_\_builtin\_expect(!!(x), 1)

6 #define unlikely(x) \_\_builtin\_expect(!!(x), 0)

7 #else

8 #define likely(x) (x)

9 #define unlikely(x) (x)

10 #endif

11

12 #endif


---

[ Stan Home Page ]

*© 2011–2014,
Stan Development Team.*
